# Anogenital Distance: Bailey and Renner Respond

**Published:** 2006-07

**Authors:** John E. Bailey, Gerald Renner

**Affiliations:** Cosmetic, Toiletry, and Fragrance Association, Washington, DC, E-mail: baileyj@ctfa.org; Colipa, The European Cosmetic Toiletry and Perfumery Association, Brussels, Belgium

In his letter, Weiss misrepresents the arguments presented in our letter (McEwen and Renner 2006) regarding the study of Swan et al. (2005). We pointed out that a value for “normal” anogenital distance (AGD) is not known and that without this information, “abnormal” AGD values cannot be determined. Swan et al. (2005) measured AGD in a limited number of subjects (134 boys) who varied widely in age, height, and weight. This small sample size is inadequate to determine a normal AGD value, and there are no historical control data for AGD in male human infants using a definition of AGD comparable to the one used by Swan et al.

Although the significance of AGD values in humans, if any, is unknown, it is clear that a meaningful study with AGD as the end point of interest requires knowledge of normal values as a prerequisite. Further, the lack of knowledge of normal AGD values is only one of the significant limitations of the study by Swan et al. (2005); others were identified in our previous letter (McEwen and Renner 2006).
